# DENV Inhibits Type I IFN Production in Infected Cells by Cleaving Human STING

**DOI:** 10.1371/journal.ppat.1002934

**Published:** 2012-10-04

**Authors:** Sebastian Aguirre, Ana M. Maestre, Sarah Pagni, Jenish R. Patel, Timothy Savage, Delia Gutman, Kevin Maringer, Dabeiba Bernal-Rubio, Reed S. Shabman, Viviana Simon, Juan R. Rodriguez-Madoz, Lubbertus C. F. Mulder, Glen N. Barber, Ana Fernandez-Sesma

**Affiliations:** 1 Department of Microbiology and the Global Health and Emerging Pathogens Institute, Mount Sinai School of Medicine, New York City, New York, United States of America; 2 Mount Sinai Graduate School of Biological Sciences, Mount Sinai School of Medicine, New York City, New York, United States of America; 3 Department of Cell Biology, University of Miami School of Medicine, Miami, Florida, United States of America; 4 School of Cellular and Molecular Medicine, University of Bristol, Bristol, United Kingdom; 5 Department of Medicine, Division of Infectious Diseases, Mount Sinai School of Medicine, New York City, New York, United States of America; 6 Center for Applied Medical Research (CIMA), University of Navarra, Pamplona, Spain; Washington University School of Medicine, United States of America

## Abstract

Dengue virus (DENV) is a pathogen with a high impact on human health. It replicates in a wide range of cells involved in the immune response. To efficiently infect humans, DENV must evade or inhibit fundamental elements of the innate immune system, namely the type I interferon response. DENV circumvents the host immune response by expressing proteins that antagonize the cellular innate immunity. We have recently documented the inhibition of type I IFN production by the proteolytic activity of DENV NS2B3 protease complex in human monocyte derived dendritic cells (MDDCs). In the present report we identify the human adaptor molecule STING as a target of the NS2B3 protease complex. We characterize the mechanism of inhibition of type I IFN production in primary human MDDCs by this viral factor. Using different human and mouse primary cells lacking STING, we show enhanced DENV replication. Conversely, mutated versions of STING that cannot be cleaved by the DENV NS2B3 protease induced higher levels of type I IFN after infection with DENV. Additionally, we show that DENV NS2B3 is not able to degrade the mouse version of STING, a phenomenon that severely restricts the replication of DENV in mouse cells, suggesting that STING plays a key role in the inhibition of DENV infection and spread in mice.

## Author Summary

Dengue virus (DENV) is a pathogen with a high impact in human health that replicates in a wide range of cells of the immune system. To efficiently infect humans, DENV must evade or inhibit fundamental elements of the innate immune system, namely the type I interferon response (IFN). Thus, DENV can inhibit type I IFN signaling (described by several groups), and type I IFN production (described by our group). We documented the inhibition of type I IFN production in human monocyte derived dendritic cells (MDDCs) with an otherwise strong cytokine and chemokine profile in those cells and that the NS2B3 protease complex of DENV functions as an antagonist of type I IFN production, and its proteolytic activity is necessary for this event. Here we identify the human adaptor molecule STING as a target of the NS2B3 protease complex and characterize the mechanism of inhibition of the type I IFN production in primary human MDDCs mediated by this viral factor. We also describe that DENV NS2B3 cannot degrade the mouse version of STING, a phenomenon that strictly restricts the replication of DENV in mouse cells, suggesting that STING plays a key role in the inhibition of DENV infection and spread in mice.

## Introduction

Viral infections have a vast impact on human health, resulting in hundreds of thousands of deaths yearly. To replicate and spread, these intracellular pathogens subvert the host cellular defense machinery. Dengue virus (DENV) is the most prevalent arbovirus in humans, and productively infects cells that are involved in the immune response, such as monocytes, B cells, macrophages and dendritic cells (DCs) among others [1], [2], [3], [4], [5]. Like most viruses, DENV has evolved in order to inhibit or evade different aspects of the innate immune system, the first line of human defense against microbes. DCs are antigen presenting cells (APCs) and some of the first cells that interact with the virus after the bite of an infected mosquito. Infection of these cells induces their activation, which results in their migration to the lymph nodes where the virus can infect other susceptible cells. The kinetics of infection of different cells in the immune system is not well documented, due to the lack of immune-competent mouse models for dengue disease. Nevertheless, in mice defective for type I IFN signaling, one of the most accepted current models for dengue disease, it has been shown that DCs and macrophages are productively infected by DENV [3], [4], [5], [6] reviewed in [7].

DENV is a single stranded RNA virus of positive polarity that, after entering the cytoplasm of the host cell, releases its genome and synthesizes a polyprotein using the cellular machinery, as a first event of the viral cycle. The DENV polyprotein is cleaved by the viral protease complex (NS2B3) and cellular proteases, including furin [8]. After this processing, some of the viral proteins have the ability to rearrange the ER membrane and create the micro-environment necessary for the production of de novo synthesized viral genomic RNA. During this event, DENV accumulates products with conserved molecular structures, like RNA with 5′-triphosphate moiety or double stranded RNA, also referred to as pathogen associated molecular patterns (PAMPs). These foreign molecules are ligands of different cellular receptors engaged in their recognition, known as pattern recognition receptors (PRRs). PRRs are mainly divided into two different classes depending on their localization, associated with either the membrane or the cytoplasm. The Toll-like receptor (TLR) family is composed of membrane proteins with domains that are designed to detect extracellular PAMPs. On the other hand, the cytosolic DExD/Hbox RNA helicase proteins that contain caspase-recruiting domains (CARDs), referred to as RIG-I and MDA-5, can detect specific PAMPs present in the cytoplasm. These last two cytoplasmic sensors together with the TLR family members (TLR3/TLR7/TLR8) have been described so far as the most relevant DENV sensors [9], [10], [11]. After recognition of the mentioned PAMPs by the C-terminal helicase domain of RIG-I and MDA-5, these undergo a conformational change that exposes their CARD domains and promote the interaction with different down-stream molecules. One of the most well studied down-stream molecules, referred as IPS-1 (also known as, MAVS, CARDIF or VISA), is located in the outer membrane of the mitochondria and transmits the signal via different molecules, including the tumor necrosis factor receptor associated factors 6 and 3 (TRAF6 and TRAF3) and the IκB kinase (IKK) family members (TBK1, IKKα, IKKβ and IKKε) among other cellular factors [reviewed in [12]]. Recently three different groups, using cDNA library screening of genes that induced the IFNβ promoter, described an adaptor protein that localizes in the endoplasmic reticulum (ER). This protein was named as stimulator of the interferon gene (STING) [13], mediator of IRF3 activation (MITA) [14] and endoplasmic reticulum IFN stimulator (ERIS) [15]. Also the same protein, referred to as MYPS, was previously identified as a mediator of anti-major histocompatibility complex II monoclonal antibody-induced apoptosis in B-lymphoma cells [16]. STING is highly expressed in several immune cells, including macrophages and DCs, as well as endothelial and epithelial cells [13]. This protein can interact with RIG-I and IPS-1, but not with MDA-5, and the signaling mediated by this adaptor is independent of the sensing by the TLR family members [17]. In two recent reports, it was documented that STING is involved in the pathway that mediates the detection of pathogens with DNA genomes [18] and has a role as a direct sensor of cyclic di-nucleotides, a signaling molecule produced exclusively by bacteria and archea [19]. Activation of STING by some of these stimuli leads to its relocalization with TBK1 from the ER to perinuclear vesicles containing the subunit of the exocyst complex 5 (Sec5) followed by the phosphorylation of TBK-1 and the subsequent activation of the transcription factors IRF3/7 and NFκB, which translocate to the nucleus and complex with ATF2/c-Jun to induce the expression of type I IFN and pro-inflammatory cytokines [17].

A remarkable hallmark of highly virulent human pathogens is the ability, acquired through evolution, to inhibit this innate immune response by the expression of viral factors that affect one or several steps of the above described signaling cascade. Some of the most notorious examples are the influenza virus NS1 protein, that targets RIG-I for degradation, minimizing the sensing of influenza virus PAMPs by this PRR [20] or the Hepatitis C virus NS34A protease complex that cleaves the adaptor IPS-1 to interrupt the signaling that ends with the activation of IRF3, NFκB and the subsequent production of type I IFN in human hepatocytes [21].

Our group has documented that DENV is a weak inducer of type I interferon in human DCs, in particular when compared with other viruses that competently produce these cytokines in large amounts, such as Newcastle disease virus (NDV) [22] and Semliki Forest virus (SFV) [23]. This lack of type I IFN production by DCs infected with DENV results in an impaired ability of those DCs to prime T cells toward Th1 immunity, an effect that can be reversed by the addition of IFNβ [24]. Nevertheless, DENV is able to induce the expression of some pro-inflammatory cytokines at early times post infection, which we hypothesize is a strategy used by this virus to attract more cells to the site of infection by allowing the expression of some chemo-attractants by infected cells. Our group described that the infection by DENV does not induce the phosphorylation of IRF3 in human primary cells, resulting in an inhibition of type I IFN production [24]. In a subsequent report, we examined the ability of DENV-infected DCs to respond to a variety of type I IFN-triggering signals using potent stimulators such as NDV, SeV, SFV, or TLR-3 ligand poly(I∶C) [25]. This effect is viral dose dependent and takes place as early as 2 hours after DENV infection. We also showed that the inhibition of IFNα/β production after NDV infection in DENV-infected DCs is not a bystander effect, implying an active role of the DENV-infected DC population in the inhibition of IFNα/β. By using an NDV vector strategy to express the individual DENV non-structural proteins (NS2A, NS2B3, NS4A and NS4B), we showed that only the recombinant NDV expressing the protease complex NS2B3 inhibited IFNα expression in infected MDDCs, as compared to NDV alone. Similar results were obtained using an IFNβ promoter activity assay in 293T cells. Catalytically inactive NS2B3 mutants showed a diminished inhibition of this phenotype, which highlighted the important role for the protease activity of the NS2B3 protein as inhibitor of the type I IFN production. Interestingly, the proteolytic core of NS2B3, consisting of the last 40 amino acids of NS2B and the first 180 amino acids of NS3, was enough to reduce the activation of the IFNβ promoter by a strong stimulus, such as Sendai virus (SeV) infection.

DENV has also been shown to express inhibitors of the type I IFN signaling cascade [26] and has been shown to encode for at least four non-structural proteins NS2A, NS4A, NS4B and NS5 that target different components of this pathway. The most remarkable example is the proteasomal degradation of human STAT2 by the NS5, a phenomenon that does not occur in mouse cells, which makes mouse STAT2 a restriction factor for DENV replication in these animals [27], [28], [29], [30], [31]. In summary, DENV can successfully inhibit two fundamental steps of the innate immune system, both the inhibition of the type I IFN production and the signaling. In this way, DENV reduces the expression of hundreds of interferon inducible genes that would otherwise establish the antiviral state and control the spread of the infection in the host.

In the present report, we describe the mechanism of inhibition of type I IFN production by DENV in primary human and mouse cells and identify the human adaptor molecule STING as a target of the DENV NS2B3 protease complex. We demonstrate that the proteolytic activity of this viral factor is crucial for the cleavage and degradation of STING and this phenomenon impairs the production of type I IFN in DENV infected cells. Furthermore, we show that DENV NS2B3 is not able to cleave the mouse version of STING. Using STING double knockout mouse embryonic fibroblast (MEFs) and human dendritic cells, we demonstrate the relevant role of this host factor in the restriction of DENV replication in mouse cells. This is the first report showing STING as a target for cleavage and degradation by a viral protein to inhibit innate immune responses and as a host restriction factor for virus infection in primary cells.

## Results

### Dengue virus NS2B3 protease complex cleaves human STING

Previous results from our laboratory showed that dengue virus inhibits type I IFN production in human primary dendritic cells and that this inhibition requires a proteolytically active NS2B3 protease complex [24], [25].

**Figure 1:**
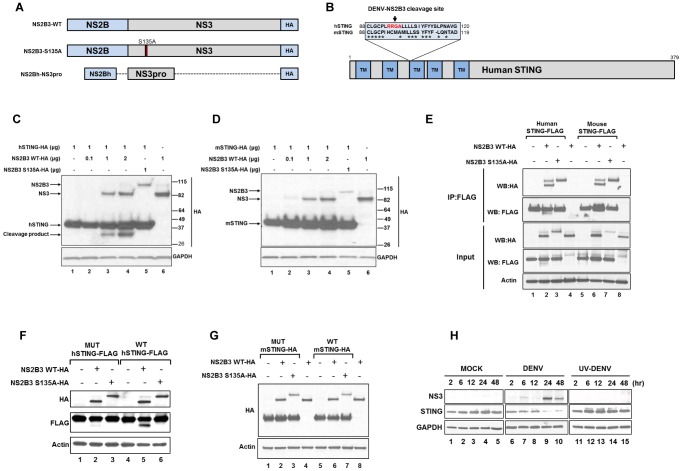
DENV NS2B3 protease complex cleaves human STING but not mouse STING. (A) Schemes of the DENV-NS2B3 protease constructs used [wild type (WT), proteolyticaly inactive mutant (S135A) and proteolytic core (NS2Bh-NS3pro)]. (B) Representative map of the human adaptor STING showing the five transmembrane domains (TM) presented at the N-terminal region. Highlighted aminoacid sequence alignment of human and mouse STING at the N-termini of TM3 (light blue), and cleavage site of DENV NS2B3 (highlighted in red). (asterisk) indicates positions which have a single, fully conserved residue. (C) Co-transfection of DENV-NS2B3 plasmid constructs and human STING expressing plasmid in 293T cells. The samples were analyzed by SDS-PAGE and detected by Western blotting (WB). (D) Same co-transfection experiment described in (C) but using mouse STING expression plasmid. (E) Analysis of the interaction between DENV-NS2B3 protease and STING by co-immunoprecipitation assay. HA-tagged NS2B3 and FLAG-tagged human and mouse STING plasmids were transfected in 293T cells. After 48 h, cells lysates were prepared and immunoprecipitation was carried out using anti-FLAG antibody. (F) Evaluation of cleavage ability of DENV-NS2B3 on WT human STING and mutant version of human STING harboring aminoacids sequence from mouse STING (RRG/HCM) by WB using anti-HA and anti-FLAG antibodies. (G) Evaluation of cleavage ability of DENV-NS2B3 on WT mouse STING and mutant version of mouse STING harboring aminoacids sequence from human STING (IHCM/LRRG) by WB using anti-HA antibodies. (H) Degradation of endogenous human STING by DENV in primary human DCs analyzed by WB of endogenous levels of human STING and DENV NS3 protein in human DCs infected with MOCK, DENV and UV-DENV at different times post infection using anti-STING and anti-NS3 antibodies described in materials and methods.

In order to identify potential NS2B3 targets we performed a bioinformatic search for potential DENV protease cleavage sites contained within members of the type I IFN pathway [32]. We identified putative cleavage sites in several known members of the type I IFN pathway (see table 1). After testing the factors shown in table 1 for their susceptibility to be cleaved by the DENV protease NS2B3, we observed that only STING was cleaved in our experimental set up (data not shown and figure 1). We have generated a wild type DENV-NS2B3, and a proteolytically inactive version (NS2B3-S135A), (Figure 1A) by direct mutagenesis [25] that were used to analyze the potential cleavage of STING by the DENV protease complex. When we compared the human amino acid sequence of human STING to its mouse counterpart we noticed that the putative NS2B3 cleavage site in hSTING which is situated at the beginning of transmembrane domain 3 (Figure 1B), is absent in mouse STING. In order to test the susceptibility of human and mouse STING proteins to proteolytic cleavage or degradation, we co-expressed a C-terminally HA-tagged STING alongside a wild type or catalytically active DENV-NS2B3, and a proteolytically inactive version (NS2B3-S135A) in 293T cells, (Figure 1A) and analyzed them by western blot (Figures 1C, 1D). In the presence of WT NS2B3 we observed the full-length 42 kDa human STING and an additional band of about 32 KD, which is consistent with a C-terminal region product of a cleavage occurring within the first 96 aa of STING (Figure 1C). The additional band was not visible when the mouse version of STING or a catalytically inactive NS2B3 was used (Figure 1C and 1D). This putative cleavage site for the DENV NS2B3 lies very close to the conserved cysteine motif C88xxC91, or redox motif, recently described to be required for dimerization of STING and subsequent signaling in the type I IFN production pathway [33]. Regardless of their susceptibility to cleavage by the DENV NS2B3 complex, both the human and mouse versions of STING co-immunoprecipitated with the WT DENV NS2B3 complex and the proteolytically inactive mutant NS2B3 S135A (Figure 1E, lanes 2, 3, 6 and 7). To map the putative cleavage site of human STING for the DENV NS2B3 complex, we mutated the sequence corresponding to the first three amino acids of the human site, RRG (shown in figure 1B as hSTING in red) with the sequence corresponding to the amino acids HCM found in the mouse version of STING (shown in figure 1B as mSTING). These recombinant versions of STING were co-transfected into 293T cells with the WT and mutant version of the NS2B3 protease and the ability of the DENV protease to cleave STING (Figure 1F, lane 5) was drastically reduced when the mouse sequence was present in hSTING (Figure 1F, lane 2). These data confirm the requirement for amino acids RRG for efficient cleavage of STING by the DENV NS2B3. However, replacement of the corresponding amino acid sequence of mouse STING (IHCM) by the human putative cleavage sequence (LRRG) does not render mouse STING susceptible to cleavage by the DENV protease (Figure 1G, lane 2), suggesting that additional flanking amino acids are required for this cleavage. Altogether, these results strongly suggest that STING is a target for NS2B3 in human cells and possibly a restriction factor for DENV infection in the mouse.

**Table 1:** List of putative targets of DENV protease.

Host Factor	Putative Cleavage site for DENV NS2B3		
IRF3	RAGQWLWA	**QR LG**	HCHTYWAVS
IRF7	LRGPQLWA	**RR MG**	KCKVYWEVG
TLR3	YKLNHALCL	**RK GM**	FKSHCILNWP
TBK1	TLLLYQELM	**RK GI**	RWLIELIKDD
IKKe	PPIAPYPSP	**TR KD**	LLLHMQELCE
RIG-I	VGNVIKMIQ	**TR GR**	GRARGSKCFL
STING	VRACLGCPL	**RR GA**	LLLLSIYFYYSL

Human factors involved in type I IFN production, harboring putative cleavage sites for DENV NS2B3 protease complex based on Li et al. 2005 [33]. Amino acids shown in bold in the middle column represent the putative target sequences for cleavage by the DENV NS2B3.

To test whether endogenous STING undergoes the same NS2B3-dependent processing as in overexpression experiments in 293T cells, we infected human MDDCs with DENV-2 (16681 strain) and analyzed the cell lysates by western blot at different time points (Figure 1H). Infection of MDDCs by DENV resulted in the degradation of STING that could be detected at 24 and 48 hours post infection (hpi) (Figure 1H, lanes 9 and 10) which correlate with peak expression levels of the NS2B3 (as detected with NS3 specific antibodies). As expected, this degradation of STING was not observed in MDDCs treated with UV-inactivated DENV or mock treated cells (Figure 1H, lanes1–5 and 11–15). These data demonstrate that DENV NS2B3-dependent cleavage of endogenous human STING occurs in cells relevant to DENV infection (MDDCs), and therefore has the potential to play a crucial role in inhibition of type I IFN production.

### DENV NS2B3 inhibits induction of IFNβ and p55-C1B promoters by human STING

**Figure 2:**
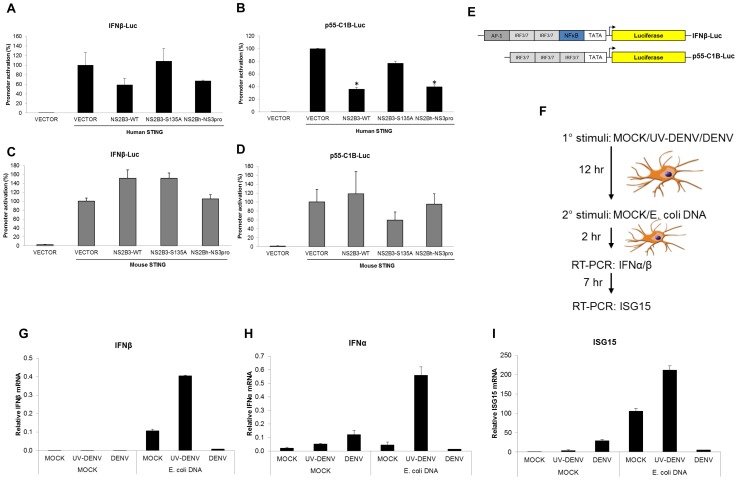
DENV NS2B3 inhibits induction of IFNβ and p55-C1B promoters by human STING. (A) 293T cells were co-transfected with a vector encoding for an IFNβ promoter driving the luciferase gene, a plasmid encoding for human STING, and plasmids for different versions of DENV protease (see Fig. 1D). Luciferase activity was measured 24 hours after transfection. (B) Same as described in (A) but using a plasmid encoding for the mouse version of STING. (C) 293T cells were co-transfected with a vector encoding for p55-C1B promoter driving the luciferase gene, a plasmid encoding for the human STING and plasmids for three different version of the DENV protease (see Fig. 1D). (D) Same as described in (C) but using a plasmid encoding for the mouse version of STING. (E) Scheme of the luciferase expressing vectors used, harboring the promoters for IFNβ and p55-CIB (F) Scheme of experimental design. MDDCs were treated with MOCK, DENV and UV inactivated DENV. After 12 hours, the three sets of cells were stimulated with MOCK or purified *E. coli* DNA and total RNA was purified at 2 and 7 hours post treatment. Relative expressions of type I IFN and ISGs, normalized against rsp11 and α-tubulin, were measured by qRT-PCR at specific time points. (G) INFβ mRNA. (H) IFNα mRNA. (I) ISG15 mRNA. Data shown represents one of two independent experiments. Error bars are the standard deviation of two individual replicates. *, p<0.05.

We next investigated whether STING cleavage by DENV NS2B3 had an impact on its ability to mediate the signaling necessary for type I IFN production. We transfected 293T cells with either hSTING (Figures 2A and 2B) or mSTING (Figures 2C and 2D) and the three different versions of the DENV protease: wild type, the proteolytically inactive version (NS2B3-S135A) and the proteolytic core (NS2Bh-NS3pro) alongside luciferase reporter constructs driven by either an IFNβ promoter (IFNβ-Luc) or by three IRF3/7 binding sites (p55-C1B-Luc) (kindly provided by Dr. Megan Shaw and shown in figure 2E schematically) [34]. As shown in figures 2A and 2B, cleavage of hSTING by the DENV NS2B3-WT and NS2Bh-NS3pro greatly inhibited its ability to activate both reporter constructs, while transfection of the mutant version of the protease did not. Conversely, the DENV NS2B3 had minimal or no impact on the ability of mSTING to induce activation of either of the reporter constructs used. We did not observe any DENV NS2B3-dependent inhibition when the human adaptor TBK1 was used as a positive control to induce the IFNβ promoter, demonstrating that the inhibition during dengue infection occurs upstream of this adaptor (data not shown). Taking together, these data demonstrate that cleavage of hSTING by the DENV NS2B3 precludes the induction of type I IFN responses. Moreover, to validate the observed results in human primary cells, we used *E. coli* DNA, a known inducer of STING signaling [35] to treat human MDDCs previously infected with DENV, UV-inactivated DENV or mock treated (as shown schematically in figure 2F). Figures 2G, 2H and 2I show that only live DENV but not UV inactivated DENV was able to inhibit the induction of IFNβ, IFNα or ISG15 by this ligand in human MDDCs.

### DENV NS2B3 antagonizes type I IFN production in human but not in mouse cells

**Figure 3:**
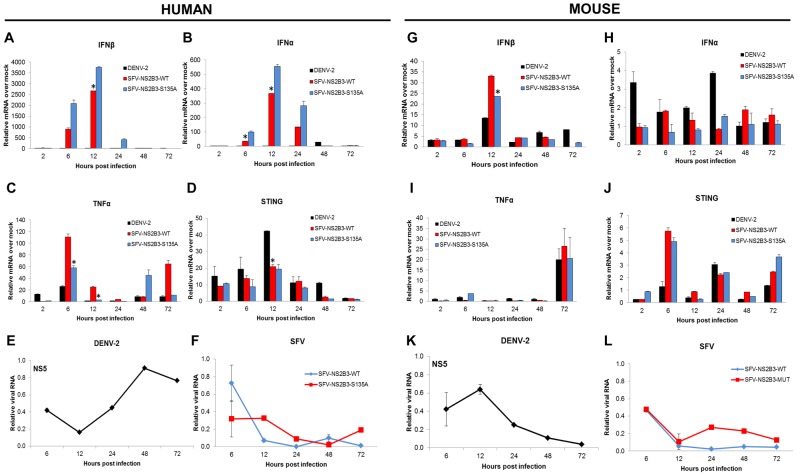
DENV NS2B3 inhibits type I IFN production in human but not in mouse dendritic cells. Human dendritic cells were infected with MOCK, DENV-2 (16681), SFV-NS2B3-WT and SFV- NS2B3-S135A using a MOI = 1, and total RNA was extracted at 2, 6, 12, 24, 48 and 72 hpi (A to F). Relative expressions of different proteins and viral RNAs were measured by qRT-PCR at specific time points normalized against rsp11 and α-tubulin. (A) INFβ mRNA. (B) IFNα mRNA. (C) TNFα mRNA. (D) STING mRNA and (E) DENV RNA, (F) SFV RNA. (G to L) Mouse dendritic cells were infected with MOCK, DENV, SFV-NS2B3-WT and SFV- NS2B3-S135A using an MOI = 1, and total RNA was extracted at 2, 6, 12, 24, 48 and 72 hpi. Relative amounts of mRNA were measured by qRT-PCR using 18S and β-actin as housekeeping genes. (G) IFNβ mRNA. (H) IFNα mRNA. (I) TNFα mRNA. (J) STING mRNA. (K) DENV RNA (L) SFV RNA. Data re representative of three independent experiments from three different donors (A to F) and two individual experiments (G to L). Error bars indicate standard deviations of the mean from duplicate samples.*, p<0.05.

To validate the results described using p55-C1B and IFNβ promoter assays in a primary cell model, we measured IFNα/β production upon infection of MDDCs either with DENV or a Semliki forest virus (SFV) expressing the DENV NS2B3 protease complex or the mutant version of the protease (NS2B3-S135A) as a control [23], [25]. Consistent with our earlier report [24], human MDDCs infected with DENV-2 (16681 strain) were unable to produce IFNα/β. Furthermore, SFV-NS2B3 induced significantly lower levels of IFNα/β mRNA than the SFV-NS2B3-S135A (Figures 3A and 3B). Interestingly, SFV-NS2B3-WT induced significantly higher expression of TNFα at early times post-infection compared to SFV-NS2B3-S135A control (Figure 3C). As shown previously, this would suggest an involvement of the NS2B3 protease complex in the expression of this pro-inflammatory cytokine [24]. As expected, the infection of MDDCs by DENV up-regulated the expression of STING in these cells (Figure 3D). Figure 3E shows the kinetics of infection by DENV in MDDCs, with the peak of viral RNA at 48 hpi. In contrast, the SFV vectors used in these studies show low levels of viral RNA at late times after treatment (Figure 3F), since these vectors are replication deficient [36]. Then we infected mouse bone marrow-DCs (BM-DCs) using the same viruses, and analyzed the gene induction profile in those cells. As expected, infection of BM-DCs by DENV was rapidly controlled, consistent with the inability of DENV to infect mouse cells, and showed an opposite kinetic of viral RNA synthesis compared to the observed pattern in human DCs, with a modest induction of cytokines (Figure 3G to 3K). SFV is an alphavirus that can replicate in mouse cells, and DCs in particular. In this context, the SFV-NS2B3 exhibited a higher induction of both IFNα/β genes compared to the SFV-NS2B3-S135A control, showing an opposite profile than that observed in human DCs, in which SFV-NS2B3-S135A induced higher levels of IFNα/β genes (Figure 3G and 3H). Again, the kinetics of infection of the SFV vectors in mouse DCs (Figure 3L) show very low levels of viral RNA, consistent with their lack of productive infection in these cells [36]. The observed phenomenon agrees with the inability of recombinant DENV-NS2B3 to cleave mouse STING and decrease the activity of the IFNβ and p55-C1B promoters induced by this adaptor protein (Figure 2C and 2D).

### STING strongly restricts the replication of DENV in mouse cells

**Figure 4:**
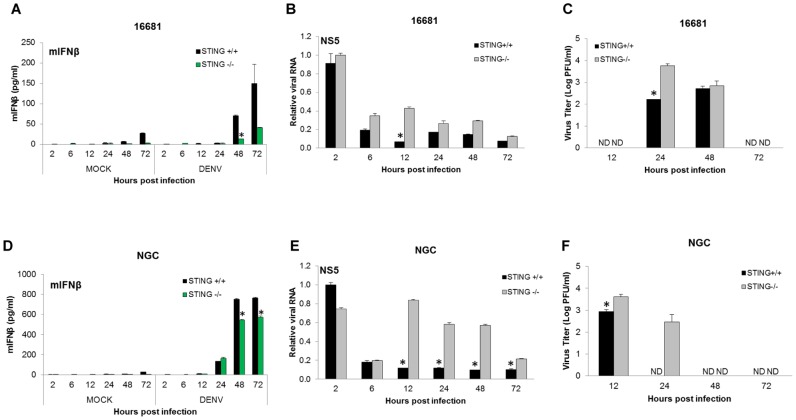
STING restricts replication of DENV in mouse cells. Sting ^+/+^ and Sting ^−/−^ MEFs were infected with DENV-2 (16681 strain) (A, B and C) and DENV-2 (NGC strain) (D, E and F) at an MOI of 5 and total RNA was extracted at the indicated times post infection. Levels of IFNβ were measured by ELISA(A and D) and DENV RNA levels were measured by qRT-PCR (B and E) using 18S and β-actin for normalization.. Infectious particles released to the supernatants of infected MEFs were measured by plaque assay on BHK cells (C and F) as described in materials and methods. Data are representative of two independent experiments. Error bars represent standard deviations of the mean of two replicate samples. *, p<0.05.

To explore STING's impact on the DENV replication in mouse cells, we used WT (*Sting ^+/+^*) and STING double knockout (*Sting ^−/−^*) mouse embryonic fibroblasts (MEFs). First, we infected WT and *Sting ^−/−^* MEFs with two different DENV-2 strains, 16681 and NGC (a strain that was obtained after several passages in mouse brain) [37], with an MOI of 5. Then, we measured the ability of the two DENV-2 strains to induce IFNβ production, to replicate in these cells and to release infectious particles from those cells (Figure 4A–4F). Both DENV-2 strains induced significantly higher levels of IFNβ in WT MEFs as compared to the *Sting ^−/−^* MEFs (Figure 4A and 4D), underlining the relevance of STING in the signaling of type I IFN upon the infection with DENV. Consistent with the observed low induction of IFNβ, *Sting ^−/−^* MEFs were permissive to DENV replication while, despite the high MOI used, replication of DENV 16681 and NGC was rapidly controlled in WT MEFs (Figure 4B and 4E). The production of infectious particles by the two DENV-2 strains in WT and *Sting ^−/−^* MEFs was measured by plaque assay and shows that the KO MEFs were more permissive to DENV infection than the WT MEFs and have very different peaks of infection (Figure 2C and 2F). To test whether our observation was independent from the high MOI and viral strains used, we repeated the infection using different DENV serotypes (DENV-2 16681 strain, DENV-3 PR-6 strain and DENV-4 H-241 strain) and an MOI of 1, with similar results (data not shown). These results are likely due to the inability of DENV to inhibit the type I IFN signaling in mouse cells and the establishment of the antiviral state [31]

### Mutation of cleavage site for DENV-NS2B3 restores the ability of STING to induce type I IFN production upon DENV infection

**Figure 5:**
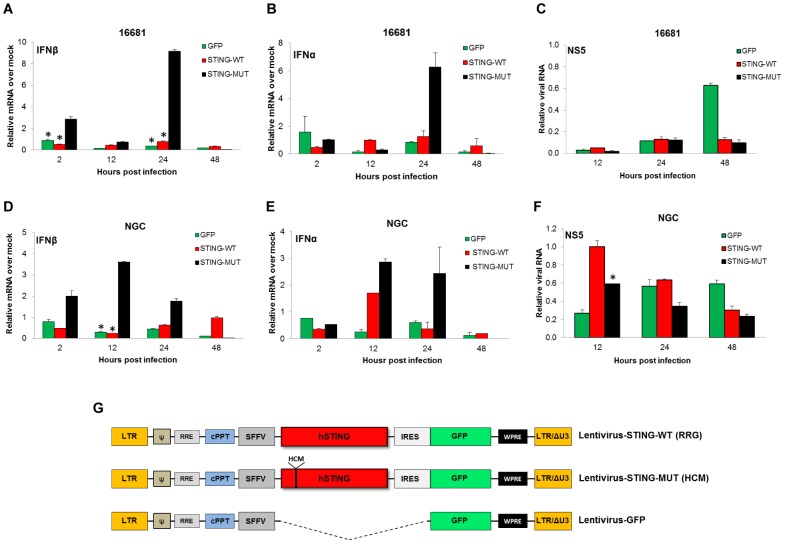
Expression of mutant STING in Sting ^−/−^ MEFs rescues their ability to produce type I IFN and to control DENV infection. Two versions of recombinant human STING (WT and MUT) and GFP control were expressed in Sting ^−/−^ MEFs using lentiviral vectors (described in materials and methods). After 24 hours, infection by lentivirus was confirmed by GFP expression and MEFs were subsequently infected with DENV-2 16681 (A–C) or DENV-2 NGC (D–F). Total RNA was extracted at the specified times post infection and the levels of IFNβ mRNA (A and D), IFNα mRNA (B and E), and DENV RNA (C and F) were measured by qRT-PCR. (G) Schematic representation of lentiviral vectors used in these experiments. Error bars represent standard deviations of the mean of two replicate samples.*, p<0.05.

To confirm the relevance of STING cleavage by the DENV-NS2B3 on the inhibition of type I IFN production upon DENV infection, we transduced *Sting ^−/−^* MEFs with lentiviruses expressing either WT human STING (STING-WT) or a mutant (uncleavable) version, that harbors the mouse STING sequence at the NS2B3-cleavage site (STING-MUT) (Figure 1B and 1F). Twenty-four hours after transduction MEFs were infected with DENV-2 at an MOI of 1 (strains 16681 and NGC) or mock treated and the levels of IFNβ, IFNα and viral RNA were measured by qRT-PCR after total RNA extraction from the cells at different times post infection (Figure 5A–5F). A schematic representation of the lentiviruses used is shown in figure 5G. For these experiments, as shown in figure 5A and 5D, MEFs expressing the uncleavable version of STING (STING-MUT) expressed significantly higher levels of IFNβ mRNA when compared to MEFs expressing WT STING (STING-WT) or to the control MEFs (GFP) confirming that the cleavage of STING by DENV-NS2B3 is necessary for the inhibition of IFNβ production in infected cells. The induction of IFNβ was detected as early as 2 hpi. Infection with the two DENV-2 strains also induced higher levels of IFNα mRNA in MEFs expressing STING-MUT than in the MEFs expressing STING-WT and the GFP control (Figure 5B and 5E). Under these experimental conditions, the replication of the mouse adapted DENV-2 (NGC) was increased in *Sting ^−/−^* MEFs expressing wild type STING as compared with STING-MUT (Figure 5F). In the case of 16681 strain, a significant increase of replication was observed only with *Sting ^−/−^* MEFs (GFP) at 48 hpi., and no significant difference was observed in MEFs expressing the two versions of STING, presumably due to a high level of lentiviral-expressed STING that could overwhelm the ability of this non mouse-adapted DENV strain to replicate in this system (Figure 5C).

### Expression of STING-MUT in primary human DCs attenuates DENV replication

**Figure 6:**
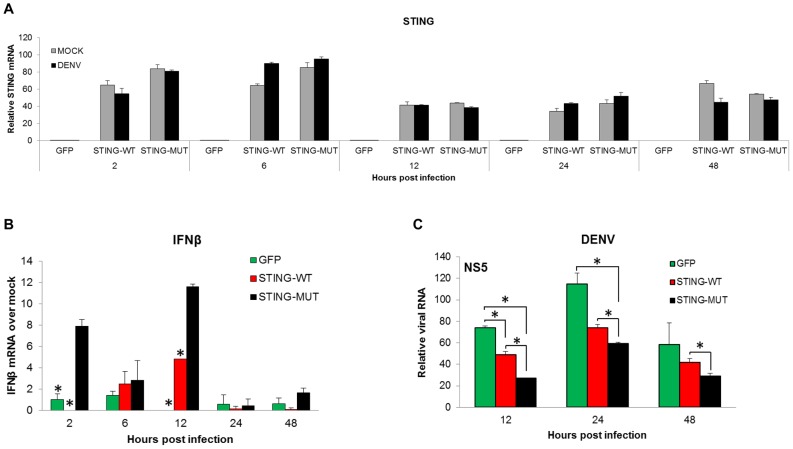
Over expression of STING in MDDCs potentiates IFNβ induction and attenuates DENV replication. Primary human monocytes were transduced with lentiviruses expressing either STING-WT, STING-MUT or GFP as a control (Figure 5G and materials and methods). After five days of culture in the presence of GMCSF and IL-4 (see materials and methods) the differentiated MDDCs, were infected with DENV-2, 16681 using an MOI of 5 and total RNA was extracted at the specified times post infection. (A) Levels of STING mRNA were measured for mock and DENV infected cells at 2, 6, 12, 24 and 48 hpi. (B) Levels of mRNA for IFNβ were measured as indicated for (A). (C) Replication of DENV as measured by qRT-PCR (materials and methods) using specific primers for DENV-2 at the specified times post infection. Data shown are representative of three individual experiments from three independent donors. Error bars represent the standard deviation of the mean of two replicate samples. *, p<0.05.

As shown in figure 5, the lack of STING cleavage was sufficient to increase the expression of type I IFN in MEFs infected with DENV. To validate these results in primary human cells, we transduced MDDCs from three different donors with the STING-expressing lentiviruses and the GFP-only control (Figure 5G). STING transduced DCs were then infected with DENV-2 at an MOI of 5 and we assessed the production of IFNβ in those cells. As shown in Figure 6A (showing one representative donor out of three), there were no significant differences between the levels of STING-WT and STING-MUT mRNA. This demonstrates that any difference in antiviral effect observed with the two different versions of STING is independent of the expression levels for these proteins. Upon DENV-2 infection, DCs expressing STING-MUT produced higher levels of IFNβ when compared with STING-WT or GFP controls (showing statistical significance at 2 and 12 hpi). The induction of IFNβ messenger RNA was detected as early as 2 hpi, which is in agreement with the results described with MEFs (Figures 4, 5 and 6B). These data confirm that detection of DENV infection by DCs takes place at early times post infection and that STING cleavage by DENV NS2B3 is fundamental to inhibit the signaling mediated by this adaptor in human cells. We next measured DENV replication kinetics and we found that viral RNA levels were significantly lower in DCs over-expressing STING-MUT when compared with STING-WT and GFP control (Figure 6C). Suggesting that the inability of DENV to cleave mutant STING and inhibit the induction of type I IFN has a direct impact on its replication kinetic and the accumulation of viral RNA in those cells.

### STING silencing in primary human cells increases DENV replication

**Figure 7:**
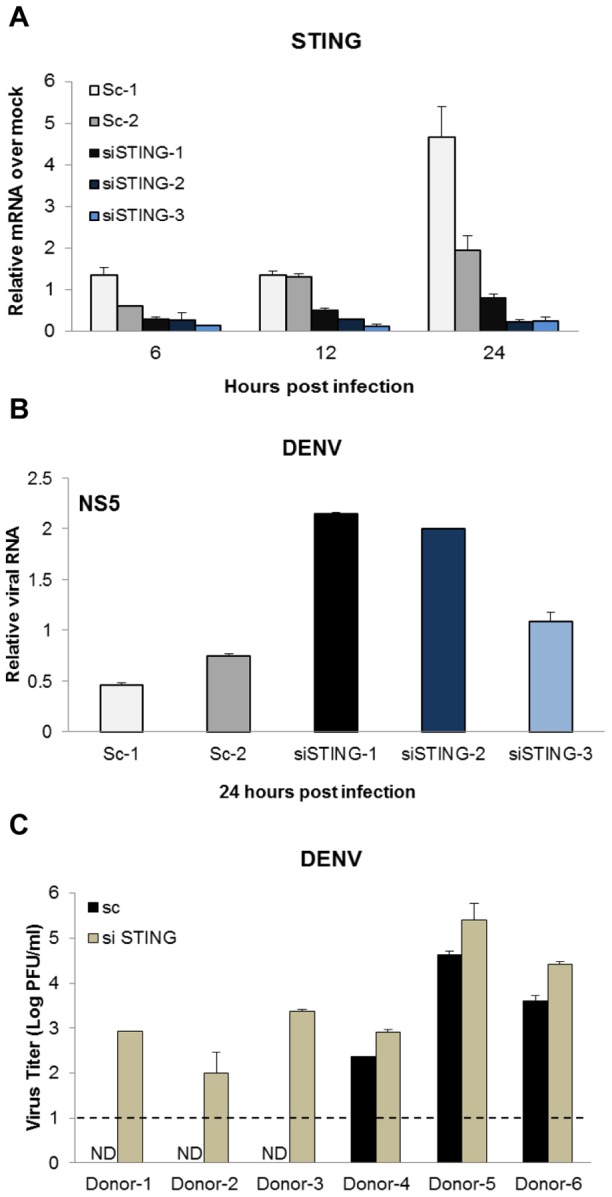
Silencing of STING in human MDDCs results in enhanced DENV replication. Primary human MDDCs were transfected with two scramble siRNAs and three STING specific siRNAs as described in the materials and methods section. 48 hours post transfection, cells were mock or DENV-2 infected (16681)at an MOI of 1. (A) Levels of mRNA for STING were quantified by qRT-PCR at the specified times post infection. (B) Levels of DENV RNA were measured at 24 hpi by qRT-PCR (data from donor 4 is shown). (C) DENV titers were determined by plaque assay of the supernatants from infected MDDCs treated with scramble and STING siRNA from six independent donors at 24 hpi (ND: not detected). Under these experimental conditions the limit of detection for the plaque assay was 10^1^ PFU (indicated with a dashed line). Data for A and B is representative of six independent donors tested in 3 separate experiments. Error bars represent the standard deviation of the mean of two replicate samples.

To determine STING's impact on DENV replication in primary human MDDCs, we used RNA interference (RNAi) to silence its endogenous expression. A decrease of STING mRNA level was observed when specific siRNAs were used compared to two scrambled control siRNAs (Figure 7A). As expected, the previously observed upregulation of STING after DENV infection was controlled by the STING siRNAs (Figure 7A). As a consequence, the reduction in STING expression resulted in an increase of DENV replication, illustrated in Figure 7B. When the viral progeny released by those infected MDDCs was quantified by plaque assay, the six donors treated with STING specific siRNA, showed viral production under this experimental conditions, however when scrambled siRNA was used, only three out of the six donors released detectable viral progeny in the supernatant (Figure 7C). Data shown in figures 7A and 7B correspond to donor 4 in figure 7C. Taken together, these data confirm that STING is a crucial restriction factor of DENV replication in human dendritic cells, since its silencing increases the levels of DENV replication in those cells.

### Several human immune cells fail to produce type I IFN after DENV infection

**Figure 8:**
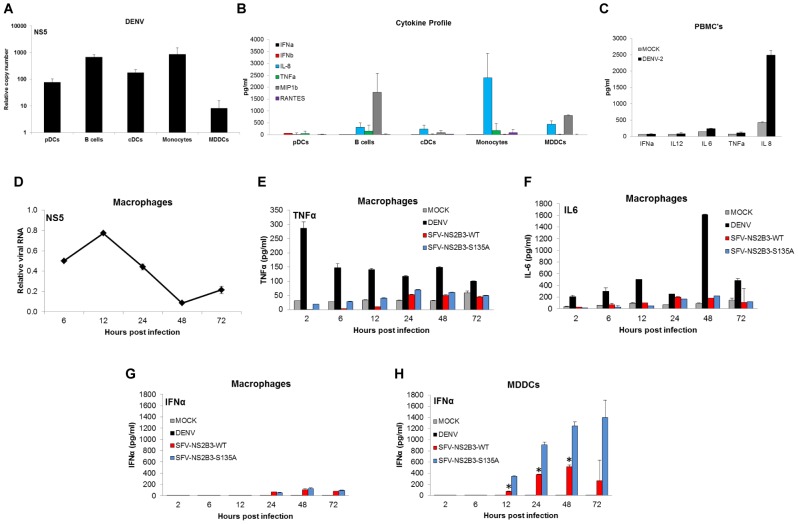
Human primary immune cells from blood fail to produce type I IFN after DENV infection. Several cell populations (pDCs, B cells, cDCs, Monocytes and MDDCs) were isolated from human blood as described in material and methods and subsequently infected with DENV-2 (166810 at MOI of 1. (A) viral RNA levels were measured in each cell population 12 h after infection by qRT-PCR using specific primers for DENV-2. (B) Cell supernatants from samples described above were collected to measure cytokine and chemokine levels by multiplex ELISA. (C) Whole PBMCs were infected with DENV-2 at MOI of 1 (black bars) or uninfected (grey bars) after ficoll centrifugation. Cytokine and chemokine levels in the PBMC supernatants were measured at 18 h post infection by multiplex ELISA. Monocyte derived macrophages (MDMs) (materials and methods) were infected with DENV-2 (16681) or SFV-NS2B3-WT and SFV- NS2B3-S135A at an MOI = 1 or mock infected for the indicated times. (D) DENV RNA levels were measured by qRT-PCR at the indicated times. Cytokines present in the supernatants from those infected MDMs were measured by multiplex ELISA: (E) TNFα levels, (F) IL-6 levels and (G) IFNα levels. (H) MDDC were infected with DENV as described for D to G and IFNα levels were measured in the supernatants by multiplex ELISA at the indicated times. Data shown for each graph are representative of three individual experiments from three independent donors. Error bars represent the standard deviation of the mean of three replicate samples.

Different populations of cells were isolated from human blood and subsequently infected with DENV-2 at MOI of 1 and 12 h after infection supernatants were collected and RNA was extracted from cells. DENV-2 RNA was detected in all cells tested including plasmacytoid DCs (pDCs), B cells, blood circulating DCs (cDCs), monocytes as well as in monocyte-derived DCs (MDDCs) (Figure 8A). We also analyzed the cytokine and chemokine expression profile in all those cells by qRT-PCR (data not shown) and by multiplex ELISA (Figure 8B) in the supernatants at 12 hpi. We observed a marked chemokine response (IL-8 and MIP1β) in monocytes, MDDCs, B cells and cDCs at this early time point, (Figure 8B). However pDCs did not show any significant chemokine profile after DENV-2 infection at this time point (Figure 8B). More interestingly, there was no significant type I IFN production observed in any of the cells tested by qRT-PCR (data not shown) or ELISA (Figure 8B). These data suggest that there is a coordinated and distinct kinetic of infection of DENV-2 in different cell populations in blood and there is a lack of type I IFN production in those cells after infection with this virus, at least at this early time point. The early time point of 12 hpi was chosen to obtain sufficient numbers of pDCs, since these cells have short half-lives and downregulate their specific cell surface markers in a rapid fashion. Nevertheless, we have previously reported that as early as 8 hpi, pDCs are able to produce type I IFN after infection with other viruses, such as NDV [24].

To rule out that the lack of type I IFN production resulted from lack of cell to cell interactions, we infected whole PBMCs with DENV-2 (MOI of 1) and 18 h after infection cell supernatants were collected. Figure 8C shows multiplex ELISA data of cell supernatants from those cultures. While there is a clear IL-8 response to DENV-2 infection in PBMCs, consistent with the strong IL-8 signature observed in sera from patients [38], there was no detectable IFNα secretion from infected PBMCs (Figure 8C). This suggests that DENV-2 may inhibit type I IFN production in susceptible cells within those cultures.

Macrophages have been shown to support DENV infection in animal models, and have been proposed to play an important role during early phases of dengue virus infection [39], [40]. We tested if monocyte-derived macrophages (MDMs) when infected with DENV were able to produce type I IFN. Figure 8D shows that macrophages are efficiently infected with DENV, with an early peak of replication and produce TNFα and IL-6 after DENV infection and after SFV expressing the WT and mutant versions of the NS2B3 DENV protease complex (Figures 8E, 8F). Under these experimental conditions we were unable to detect IFNα released by macrophages after DENV infection. Interestingly, when compared to MDDCs infected with the same viruses (Figure 8H), macrophages produce at least 10-fold lower levels of IFNα after SFV infection, and the inhibitory effect of the DENV protease in this system was less apparent (8G and 8H).

## Discussion

Activation of innate immunity due to the detection of viral replication products in the cell leads to the expression of hundreds of antiviral genes that controls the spread of the infection [12]. The inhibition of different steps implicated in these molecular pathways by viruses has been a matter of extensive study for several years. It has been demonstrated by others and by our group that DENV can inhibit both the production and signaling of type I IFN by the expression of viral proteins. In this way, DENV can mitigate the immune response induced by the host upon infection [24], [25], [29]. Here we have identified the human adaptor molecule STING as a protein with a predominant role in the recognition of DENV by the innate immune system. This adaptor protein was described to reside in the ER, a cellular organelle intimately related to the DENV replication process. Also, STING has been described as part of the TRAP (translocon associated protein) complex that can associate with RIG-I and IPS-1, two proteins with relevant roles in viral detection [41]. Ishikawa et al. also described an inhibition of the STING mediated IFNβ production by the yellow fever virus (YFV) NS4B [42]. However, when we tried to replicate these results using the DENV NS4B, this viral protein was unable to decrease the induction of luciferase mediated by STING in an IFNβ promoter assay (data not shown).

By co-expression experiments of human STING with the DENV NS2B3 protease complex we observed a specific cleavage at (94-RRGA-99), a site described as a putative target for DENV NS2B3 [32] that generated a cleaved band of approximately 32 KDa (Figure 1C and 1F). Interestingly, by analysis of the sequence alignment between human STING and its mouse version we, observed a drastic difference in the amino acid sequence in this region (94-HCMA-99) (shown in figure 1B) and the inability to cleave the mouse STING by the DENV NS2B3 was confirmed by co-expression experiments (Figure 1D and 1G). Furthermore, the impact that the STING cleavage by NS2B3 had on the signaling of IFNβ production pathway was subsequently demonstrated using IFNβ and p55-C1B promoter systems (Figure 2). In these experiments, a reduction in luciferase induction was only observed for human STING, suggesting that the cleavage confirmed by WB (showed in Figures 1C and 1F) impaired the ability of this adaptor to induce IFNβ. Recently, Jin et al. described a series of mutations in hSTING that were implicated in the activation/dimerization and subsequent induction of interferon. Interestingly, two cysteines located at C88XXC91 were fundamental for the proper induction of type I IFN after stimulation [33]. It could be interesting to investigate the presence of mutations at the cleavage site of STING for DENV NS2B3 in the human population, to identify a natural resistance to DENV infection.

While this manuscript was under review, Yu and colleagues reported by overexpression experiments that the DENV protease can cleave the adaptor molecule MITA, [43]. In the present report we provide important data on the role of this adaptor molecule in primary human and mouse cells and during the context of DENV infection. We also confirmed the cleavage and degradation of STING by the DENV NS2B3 protease in human MDDCs in the context of DENV infection, since it is important to validate these findings in a relevant primary cell system and during virus infection (Figure 1H). In primary human cells as well as in mouse cells, such as MEFs and DCs, we also found that the presence of human STING allowed for greater DENV replication and the presence of mouse STING seemed to restrict DENV replication (Figure 3, figure 4 and figure 7). We also show that the NS2B3 protease of DENV has specificity for the human STING and not for the mouse homologue of this protein (Figure 5 and figure 6), suggesting that STING may be an important restriction factor in mice.

Several viral proteases have been described as proteins that modulate cellular pathways, allowing many viruses to modify the intra and extracellular environment to promote optimal conditions for replication and spread. Some of the most remarkable characteristics observed at early times after infection by DENV are the lack of IFNα/β induction and a robust induction of pro-inflammatory cytokines like TNFα [24], [25]. As it was described in our previous work, DENV infection can abrogate IRF3 phosphorylation, but has no impact on NF-kB activity [25]. Using recombinant viruses expressing DENV-NS2B3 we observed a clear effect on the induction of TNFα in human DCs, similar to that observed with DENV infection (Figure 3 and figure 8). Also, the inhibition of luciferase activity driven by p55-C1B promoter was considerably more efficient when compared with IFNβ-promoter, since p55-C1B only harbors sites for IRF3/7 transcription factors, and IFNβ-Luc has also has response elements for NF-kβ and AP-1 transcription factors (Figures 2B and 2D). Furthermore, Ishikawa et al. overexpressed STING in 293T cells in the presence of different promoters driving the luciferase gene. Interestingly, STING stimulated IFNβ promoter up to 400-fold, IRF3 response element (PRDIII-I-Luc) up to 1,000-fold, and NF-kβ responsive promoter (NF-kβ-Luc) only up to 12-fold [41]. This observation suggested that STING is fundamentally involved in phosphorylation of IRF3, and under these experimental conditions showed a 100 fold less influence on NF-kβ induction. Taken together, these observations suggest that DENV NS2B3 protease inhibits IFNβ production by cleavage of the adaptor STING without modifying the observed NF-kβ activity induced after infection by DENV. Further work exploring the impact that DENV-NS2B3 has on the induction of NF-kβ activity in infected cells would confirm a putative role of this viral factor in the modulation of innate immune response by the induction of pro-inflammatory cytokines, a hallmark phenomenon observed during infection by DENV [44].

It is becoming increasingly clear that STING is a crucial adaptor in immune cells after infection with different viruses, such as HIV and DENV, among others [35], [45], [46]. These viruses require activation of their target cells in order to establish infection, or in the case of DENV to induce viremia in the host. Nevertheless, all viruses need to limit or inhibit the production of type I IFN in infected cells to avoid the establishment of an antiviral state in those cells. STING could be instrumental in these types of virus infections, since it can discriminate between the induction of type I IFN and the activation of the NFkB pathway. Along those lines, this report shows a novel mechanism of inhibition of IFN production by an RNA virus, namely DENV targeting STING. By inhibiting only type I IFN but not the NF-κB pathway, DENV induces a specific profile in infected human MDDCs and other susceptible primary cells that allow the virus to efficiently reach the lymph nodes and spread in the infected host, culminating in the production of viremia. All our experiments were performed in the context of primary infections with DENV, since we believe that the early events in primary infections dictate the quality of adaptive immune responses and the outcome of the infection. By targeting DCs and inhibiting the production of type I IFN in those cells, DENV may be able to efficiently modulate the generation of adaptive immune responses and establish infection in the host [24], [25].

It has been proposed that during DENV infection IPS-1 may be responsible for controlling early viral replication and type I IFN production [47], while the IFN signaling pathway (JAK/STAT) may control late viral replication and type I IFN production in DENV infected cells [48]. It is possible that interactions between STING and IPS-1 [46] may be disrupted by the DENV NS2B3 targeting of STING. This mayinhibit type I IFN production early during DENV infection in susceptible cells, although the NS2B3 has not been shown to directly interact with IPS-1. Further experiments are required to understand these complex interplays between different signaling molecules in human primary cells infected with DENV.

Our experiments demonstrate that primary human cells implicated in dengue virus infection, such as dendritic cells, macrophages, monocytes and B cells can support DENV replication, although at different levels. Interestingly, DENV infection did not induce type I IFN production in any of those human primary cells tested (Figure 8). These different blood cells may play different roles during DENV infection in humans, such as being involved in the initial infection or in the final stage of viremia. Also, since the mouse models that support DENV replication and recapitulate dengue symptoms are deficient in type I IFN responses or are reconstituted with human immune cells [6], [49], the inhibition of type I IFN in infected cells seems to be crucial for the establishment of infection by DENV. Our data on different cells from blood also show that the inhibition of type I IFN production by DENV is not a DC specific phenomenon (Figure 8).

The inability of DENV to replicate in wild type mouse cells is well documented, and many attempts have been made to develop a competent animal model to study DENV infection [50]. The data presented in this manuscript, showing the ability of DENV to replicate in *Sting ^−/−^* MEF (Figure 4), open new approaches to develop a mouse model to study DENV infection and also highlights the requirement that type I IFN production has on the innate immune system and for the control of invading pathogens. Ashour et al. described the adaptor STAT2 as a restriction factor for DENV replication in mouse cells [31]. Based on our combined data, an interesting approach would be the development of a transgenic mouse model with humanized STING and STAT2. This approach could provide an immune competent mouse model for DENV that eliminates two of the potential bottlenecks that exist for DENV replication in mice.

## Materials and Methods

### Ethics statement

The animal protocol used in this study was reviewed and approved by the University of Miami Institutional Animal Care and Use Committee (IACUC) under IACUC protocol 11–181 “Host Defense and the Regulation of Interferon Production: STING.”

The University of Miami has an Animal Welfare Assurance on file with the Office of Laboratory Animal Welfare (OLAW), National Institutes of Health. The assurance number is #A-3224-01, effective July 11, 2007. Additionally, as of July 20, 2010, the Council on Accreditation of the Association for Assessment and Accreditation of Laboratory Animal Care (AAALAC International) has continued the University of Miami's full accreditation.

### Cell lines

Vero, 293T and mouse embryonic fibroblast (MEFs), were cultured in Dulbecco's modified essential medium (DMEM) supplemented with 10% fetal bovine serum (FBS). Baby hamster kidney cells (BHK) were grown in Glasgow minimal essential medium (MEM) supplemented with 10% FBS, and 20 mM HEPES. Mosquito cells derived from *Aedes albopictus*, clone C6/36, were expanded at 33°C in RPMI medium with 10% FBS. All media were supplemented with 100 U/ml of L-glutamine and 100 µg/ml of penicillin-streptomycin. All tissue culture reagents were purchased from Invitrogen.

### Viruses

Dengue virus serotype 2 (DENV-2) strains 16681 and New Guinea C were used in this study. DENV was grown in C6/36 insect cells for 6 days as described elsewhere [51]. Briefly, C6/36 cells were infected at a multiplicity of infection (MOI) of 0.01, and 6 days after infection, cell supernatants were collected, clarified, and stored at 80°C. The titers of DENV stocks were determined by limiting-dilution plaque assay on BHK cells [52]. Semliki Forest virus (SFV) expressing GFP and DENV-NS2B3 were generated as described previously [36] and titrated in BHK cells by immunofluorescence [53].

Lentiviral vector constructs were built using conventional molecular biology techniques. Briefly, human STING cDNA was PCR amplified from pcDNA3.1 hSTING [13] and cloned into a lentiviral vector derived from pHR SIN CSGW [54] (Figure 5G). Mutations in the NS2B3 cleavage site at positions 94–96 of hSTING were obtained by overlap PCR. Residues RRG were changed to the corresponding murine sequence HCM.

Lentiviral vector derived viruses were obtained by transfection of HEK 293T with 3 plasmids encoding STING, HIV-1 Gag-Pol, and VSV-G respectively [55]. Viral supernatants were harvested 48 and 72 hours post-transfection, 0.45 µm filtered, concentrated at 14,000 g for 6 hours over a 20% sucrose cushion and frozen at −80°C until used.

### Isolation of different populations of cells from blood

Monocytes, pDCs, B cells and circulating CD11c^+^ DCs (cDCs) were isolated from blood of healthy donors (New York Blood Center) using Miltenyi isolation kits. CD14^+^ clinimacs, for monocytes, CD123/BDCA4 kit for pDCs and BDCA1 kit for cDCs. B cells were isolated as part of the BDCA1 kit for isolation of cDCs according to manufacturers' instructions. The purity of each cell population was tested by flow cytometry as described below and was routinely 85–95% for CD14^+^ cells, 87–90% for pDCs, and 95–99% for both MDDCs and cDCs.

### Infection of PBMCs or individual populations of cells from blood

Samples of 5×10^5^ isolated cell populations were infected with DENV-2, 16681 at the indicated MOI in a total volume of 100 µl of DC media for 1 hour at 37 C. Then, DC media supplemented with 4% HS was added up to a final concentration of 10^6^ cells/ml and cells were incubated for the remainder of the infections at 37 C. At the indicated times, cell supernatants were collected and cell pellets were used for RNA extractions. Whole PBMCs were used after ficoll centrifugation and samples of 60×10^6^ PBMCs were infected with DENV-2 at MOI of 1 or left uninfected. After 1 hour DC media 4% HS was added. Eighteen hpi supernatants were collected and isolation of the different cell populations after DENV-2 infection was carried out as described above.

### Generation of monocyte-derived dendritic cells (MDDCs) and monocyte derived macrophages (MDMs)

Human MDDCs were obtained from healthy human blood donors (New York Blood Center), following a standard protocol as previously described [24] and described above. Briefly, after Ficoll-Hypaque gradient centrifugation, CD14+ cells were isolated from the mononuclear fraction using a MACS CD14 isolation kit (Milteny Biotec) according to the manufacturer's directions. CD14+ cells were then differentiated to naïve DCs by incubation during 5 to 6 days in DC medium (RPMI supplemented with 100 U/ml L-glutamine, 100 g/ml penicillin-streptomycin, and 1 mM sodium pyruvate) with the presence of 500 U/ml human granulocyte-macrophage colony-stimulated factor (GM-CSF) (PeproTech), 1,000 U/ml human interleukin 4 (IL-4) (PeproTech), and 10% FBS (Hyclone). To generate MDMs, monocytes were cultured in the presence of 2000 U/ml human granulocyte-macrophage colony-stimulated factor (GM-CSF) for 10 days, and media was replenished (with same concentration of GMCSF) at days 2, 5 and 8. The purity of each cell population was confirmed by flow cytometry analysis and was at least 99% for MDDCs and 95% for MDMs.

### Generation of mouse bone marrow-derived dendritic cells

Femurs and tibia of wild-type C57BL/6 mice (Jackson) were soaked in 70% ethanol, washed with RPMI (Invitrogen), and epiphyses were cut to expose the bone marrow. The bones were flushed with RPMI supplemented with 10% fetal bovine serum (FBS) to extract the bone marrow. Cells were pelleted by centrifugation, washed once with RPMI and resuspended in ammonium chloride red blood cell lysis buffer. RBC lysis was performed for 1 minute at room temperature, stopped with RPMI-FBS and cells were collected by centrifugation. Bone marrow cells were seeded in 6-well dishes in RPMI containing 10% FBS, 50 U/ml Penicillin (Invitrogen), 50 µg/ml Streptomycin (Invitrogen), 20 ng/ml GM-CSF (Peprotech), 10 ng/ml IL-4 (eBioscience), and 40 µM beta-mercaptoethanol (BIO-RAD). Cells were cultured for 5 days at 37 degrees C, 5% CO2 and fresh media was added every 2–3 days.

### Dengue infection of MDDCs

Human and mouse DCs were obtained as described above, and at day 5 of culture, samples of 1×10^6^ cells were resuspended in 100 µl of DC-medium and were infected for 45 min at 37°C with the indicated MOI of virus (diluted in DC media) or with DC medium (mock group) in a total volume of 200 µl. After the adsorption period, DC medium supplemented with 10% FBS was added up to a final volume of 1 ml, and cells were incubated for the appropriate time at 37°C.

### siRNA transfection and Dengue infection of human MDDCs

2.5×10^4^ MDDCs were seeded per well in 96 well plates and transfected with the corresponding siRNA using the StemFect RNA transfection kit (Stemgent), according to the manufacturer instructions. Chemically synthesized 27mer siRNA duplexes were obtained from OriGene Technologies, Inc. The sequences of the STING siRNA oligonucleotides used in this study are as follows:

siSTING -1:5′-rGrGrCrArUrGrGrUrCrArUrArUrUrArCrArUrCrGrGrArUAT-3′.

siSTING-2:5′-rArCrCrUrGrUrGrArArArUrGrGrGrArUrCrArUrArArUrCAC-3′.

siSTING-3:5′-rGrGrArUrUrCrGrArArCrUrUrArCrArArUrCrArGrCrArUTA-3′.

Two random non-coding control siRNA were used: Sc-1 (5′-rUrArCrGrUrArCrUrArUrCrGrCrGrCrGrGAT-3) from Qiagen, and Sc-2 (universal scrambled negative control siRNA duplex SR30004) from OriGene Technologies, INC. 48 h after transfection, cells were infected with Dengue virus at an MOI of 1. Briefly, cells were centrifuged (400×g, 10 min), the media was removed and 25 µl of RPMI containing the appropriate amount of virus was added and the plates were incubated for 45 min at 37°C. Then, 75 µl of RPMI with 10% FBS were added and cells were incubated at 37°C for the indicated hours. Subsequently, cells were recovered by centrifugation for 10 min at 400×g, and the cell pellets were lysed for RNA isolation.

### Transduction of human MDDCs

Plated monocytes were transduced as previously described [56]. In brief, freshly isolated monocytes were transduced with VSV-G pseudo-typed SIV VLPs and each lentiviral vector construct for 3 h by spinoculation, in the presence of 2 µg/mL polybrene (Sigma). Subsequently, cells were washed, resuspended in regular growth medium described before for the generation of monocytes derived human dendritic cells and incubated for 5 days at 37°C until stimulation. At day 5 post-transduction, MDDCs were infected with DENV as described before, and cell pellets were collected at the indicated time points, and lysed for RNA isolation.

### Reporter analysis

293T cells were transfected by using Lipofectamine 2000 reagent (Invitrogen) according to the manufacturer's protocol. A type I IFN production antagonist assay was performed as described previously [25] using IFNβ-Luc and p55-C1B-Luc [34], [57]. 293T cells seeded on 24-well plates were transiently transfected with 50 ng of the luciferase reporter plasmid together with a total of 400 ng of various expression plasmids or empty control plasmids. As an internal control, 50 ng pRL-TK was transfected simultaneously. Then, 24 or 48 h later, the luciferase activity in the total cell lysate was measured by using the Dual-Luciferase Reporter Assay System (Promega) according to the manufacturer's directions.

### Western blot analysis

Transfection of 293T cells and infection of human DCs was performed as described above. Cell lysates were obtained after incubation of cells with RIPA lysis buffer (Sigma Aldrich) supplemented with complete protease inhibitor (Roche) and resuspended in a total of 50 ml of Laemmli sample buffer (Bio-Rad). Crude lysates were either boiled for 10 min or incubated at 42°C for 20 min and then kept on ice. Each sample was loaded in a polyacrylamide-SDS gel, and the proteins were electrophoretically separated by conventional methods. Proteins were transferred to nitrocellulose, and blots were incubated with anti-HA, anti-FLAG, anti-Actin anti-GAPDH (Sigma Aldrich) and rabbit polyclonal antibodies anti-hSTING [41] and anti-DENV NS3 (kind gift of Dr. Andrea Gamarnik), and developed using SNAP ID detection system (Millipore), following the manufacturer's instructions. Antibody-protein complexes were detected using a Western Lighting chemiluminescence system (Perkin Elmer).

### RNA isolation

RNA from different cells was extracted using Trizol (Invitrogen), followed by a treatment with DNase using DNA-free Ambion. The concentration was evaluated in a spectrophotometer at 260 nm, and 500 ng of RNA were reverse transcribed using the iScript cDNA synthesis kit (Bio-Rad) according to the manufacturer's instructions.

### qRT-PCR

Evaluation of the expression of human and mouse cytokines from different cell types and viral RNA was carried out using iQ SYBR green Supermix (Bio-Rad) according to the manufacturer's instructions. The PCR temperature profile was 95°C for 10 min, followed by 40 cycles of 95°C for 10 s and 60°C for 60 s. Expression levels for individual mRNAs were calculated based on their CT values using two different housekeeping genes (human: rps11 and α-tubulin genes) and (mouse: S18 and β-Actin) to normalize the data.

### Statistical analysis

One paired two tailed Student's t-test was used to analyze data. Data considered significant demonstrated p values less than 0.05.
